# Supplementary figures and images for: Male cuticular pheromones stimulate removal of the mating plug and promote re-mating through pC1 neurons in Drosophila females
Source: eLife. 2024 Sep 10;13:RP96013. doi: 10.7554/eLife.96013 (PMC11386958; doi:10.7554/eLife.96013)

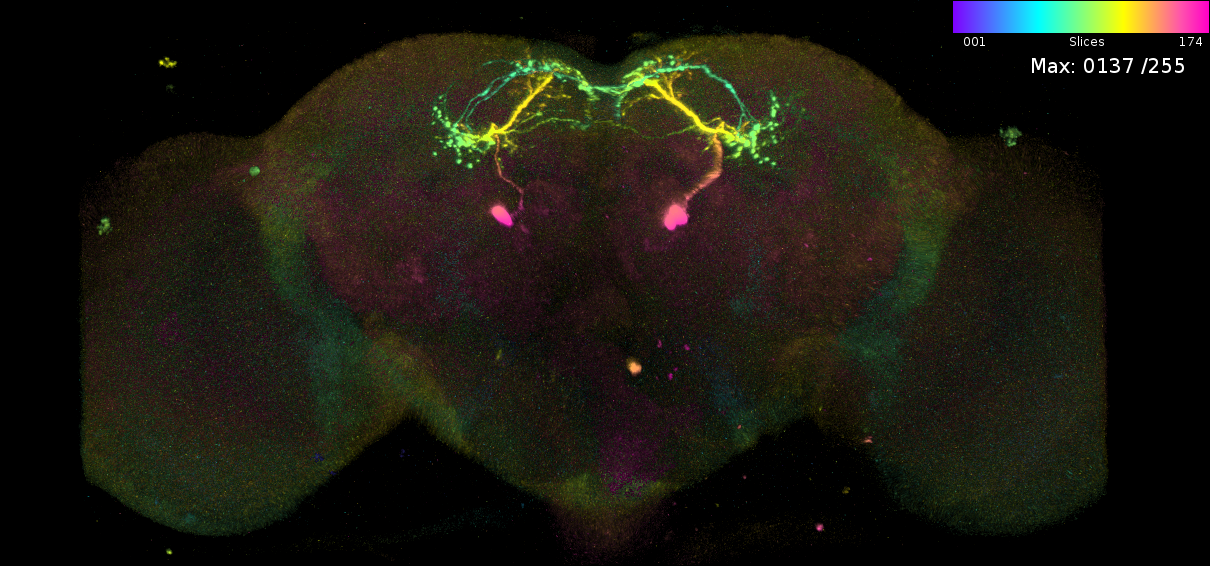

Supplement: Figure 5—figure supplement 1—source data 1. [file elife-96013-fig5-figsupp1-data1.zip › Fig5-S1C_CDMIP_pC1aSS.png]
